# Correction: High-performance liquid chromatographic method for determination of voriconazole in pure and gel formulation

**DOI:** 10.1371/journal.pone.0330554

**Published:** 2025-08-19

**Authors:** Samina Sheikh, Anwar Ejaz Beg, Mirza Tasawer Baig, Sadaf Ibrahim, Ambreen Huma, Aisha Jabeen, Zubair Anwar

The authors Mirza Tasawer Baig and Zubair Anwar should be noted as shared co-corresponding authors on this work. Mirza Tasawer Baig’s email address is: tasawer.baig@zu.edu.pk.

One of the email addresses for the corresponding author, Zubair Anwar, is incorrect. The correct email addresses for Zubair Anwar are: zubair_ana@hotmail.com and zubair_ana@baqai.edu.pk.
