# Supplementary material for: High-performance liquid chromatographic method for determination of voriconazole in pure and gel formulation
Source: PLoS One. 2024 Dec 16;19(12):e0315704. doi: 10.1371/journal.pone.0315704 (PMC11649096; doi:10.1371/journal.pone.0315704)
Supplement: Attachment — Submitted filename: review comments.docx [file pone.0315704.s001.docx]

I strongly recommend rejecting the manuscript considering missing novelty in the manuscript. There are so many reported methods available on determination of Voriconazole. Few of them are mentioned below for your reference:

<https://www.ncbi.nlm.nih.gov/pmc/articles/PMC2866341/>

<https://dergipark.org.tr/tr/download/article-file/166318>

https://www.sciencedirect.com/science/article/abs/pii/S0039914007000021
